# Role of Carbonic Anhydrases and Inhibitors in Acid–Base Physiology: Insights from Mathematical Modeling

**DOI:** 10.3390/ijms20153841

**Published:** 2019-08-06

**Authors:** Rossana Occhipinti, Walter F. Boron

**Affiliations:** 1 Department of Physiology and Biophysics, Case Western Reserve University School of Medicine, Cleveland, OH 44106, USA; 2 Department of Medicine, Case Western Reserve University School of Medicine, Cleveland, OH 44106, USA; 3 Department of Biochemistry, Case Western Reserve University School of Medicine, Cleveland, OH 44106, USA

**Keywords:** CO_2_, pH, HCO_3_^−^, facilitated diffusion, buffering, cell membranes, renal proximal tubules, red blood cells, alveoli, gas exchange

## Abstract

Carbonic anhydrases (CAs) catalyze a reaction fundamental for life: the bidirectional conversion of carbon dioxide (CO_2_) and water (H_2_O) into bicarbonate (HCO_3_^−^) and protons (H^+^). These enzymes impact numerous physiological processes that occur within and across the many compartments in the body. Within compartments, CAs promote rapid H^+^ buffering and thus the stability of pH-sensitive processes. Between compartments, CAs promote movements of H^+^, CO_2_, HCO_3_^−^, and related species. This traffic is central to respiration, digestion, and whole-body/cellular pH regulation. Here, we focus on the role of mathematical modeling in understanding how CA enhances buffering as well as gradients that drive fluxes of CO_2_ and other solutes (facilitated diffusion). We also examine urinary acid secretion and the carriage of CO_2_ by the respiratory system. We propose that the broad physiological impact of CAs stem from three fundamental actions: promoting H^+^ buffering, enhancing H^+^ exchange between buffer systems, and facilitating diffusion. Mathematical modeling can be a powerful tool for: (1) clarifying the complex interdependencies among reaction, diffusion, and protein-mediated components of physiological processes; (2) formulating hypotheses and making predictions to be tested in wet-lab experiments; and (3) inferring data that are impossible to measure.

## 1. Introduction

Carbonic anhydrases (CAs) are ubiquitous metalloenzymes that catalyze one of the most important reactions in life: the interconversion of carbon dioxide (CO_2_) and water (H_2_O) to bicarbonate (HCO_3_^−^) and protons (H^+^). Because this reaction is so fundamental to life, understanding the physiological role of this enzyme has been the focus of research for almost 90 years since the discovery of CA, in red blood cells (RBCs), by Meldrum and Roughton [1]. The discovery of CA occurred at a time during which physiologists were intensely interested in the chemical nature of blood, and in understanding how the slow interconversion of CO_2_ and HCO_3_^−^ in vitro (>1 min) could be compatible with the relative fast pulmonary capillary transit time (~1 s). Our understanding has increased substantially since those days.

We now know that CAs fall into at least seven unique families (α, β, γ, δ, ξ, η and θ)—encoded by seven evolutionarily distinct gene families—and that at least one CA family is present in virtually every living organism.

The initial descriptions of the η and θ gene families are only a few years old [2,3]. For a review of the CA families, see a recent monograph [4]. To date, investigators have deposited in the PDB over 900 CA structures [5]. Although most of these structures represent the α-family, the structures also include those for the β, γ and ξ families. For additional information on the history and recent advancements of crystallography of CA, we refer to a recent review [5].

The α-family is the most widely studied because of its role in human physiology and pathophysiology. The α-CA family comprises 16 members in vertebrates, each with a characteristic tissue-specific expression, cellular and sub-cellular localizations, kinetics and sensitivity to inhibitors [6,7,8,9]). Based on their sub-cellular localization, the members of the α-CA family can be divided into four groups: cytosolic (CA I, CA II, CA III, CA VII, CA VIII, CA X, CA XI, and CA XIII), mitochondrial (CA VA and CA VB), secretory (CA VI), and membrane-associated (CA IV, CA IX, CA XII, CA XIV, and CA XV). The mature membrane-associated CAs can be either glycosylphosphatidylinositol (GPI)-linked (CA IV and CA XV) or transmembrane (CA IX, CA XII and CA XIV).

Three of the cytosolic CAs—CA VIII, CA X and CA XI—are catalytically inactive, due to the absence of critical amino acids (e.g., His residues necessary to coordinate Zn^2+^ at the reaction center). Hence, these CAs are called CA-related proteins (CARPs). Although the CARPs are linked to human disease and are known to interact with cytosolic proteins, their physiological roles remain unclear (for reviews, see [10,11,12,13]). For all three CARPs, mutations that partially restore the catalytic site to resemble that of the classical α-CAs engender catalytic activity [14,15,16]. In addition to the 16 aforementioned CAs, two receptor protein tyrosine phosphatases (RPTPs), RPTPγ and RPTPζ, have extracellular CA-like domains (CALDs) that are closely related to the CARPs. Recent work indicates that RPTPγ may be a dual sensor for extracellular CO_2_ and HCO_3_^−^ that is responsible for the physiological response of renal proximal tubules to changes in blood levels of CO_2_ and HCO_3_^−^ [17]. It may be that, rather than interconverting CO_2_ and HCO_3_^−^, the CALD domains (and the closely related CARPs), sense CO_2_ vs. HCO_3_^−^.

Because CAs catalyze a reaction that is so fundamental in life, these enzymes affect a wide range of physiological processes in a variety of tissues and cellular compartments. These processes include respiration (e.g., transmembrane CO_2_ movements, O_2_ exchange in red blood cells via the pH and CO_2_ Bohr effects), transepithelial fluid secretion, transepithelial acid–base transport (e.g., gastric-acid secretion and pancreatic HCO_3_^−^ secretion), and acid secretion by osteoclasts in bone resorption [18,19,20,21,22]. CA-dependent acid–base transport by renal epithelia plays a central role in regulating whole-body acid–base balance, which in turns affects cell pH, the stability of which is critical for countless biochemical reactions (e.g., gluconeogenesis, ureagenesis, and lipogenesis) and nearly every other cellular function. Overexpression of CA is associated with a variety of pathological states, including glaucoma, tumorigenesis, obesity, calcification and epilepsy [9,23,24,25,26,27,28]. Thus, investigators continue to invest considerable attention to the design of CA-selective inhibitors as potential therapeutic agents [29,30,31,32].

CAs have been the subject of numerous reviews and monographs of a general nature [4,8,33,34,35,36,37,38,39,40], or that focus on specific aspects of CA biology, including diseases [41,42,43,44,45,46,47,48], protein structures [5,7,49], and drugs [8,29,30,31]. The present review differs because it focuses on the mathematical modeling of CA biology in the context of acid–base physiology and the movements of CO_2_ across cell membranes that separate the many compartments within the body. One major compartment is the extracellular fluid (ECF) that bathes the cells of the body. This is Claude Bernard’s “milieu intérieur”, and includes blood plasma, interstitial fluid, and transcellular fluid (e.g., cerebral spinal fluid). A complication is that the composition of the ECF varies regionally. Thus, blood-plasma composition not only changes as blood courses along a capillary, but the profile potentially differs among each of the body’s 10 billion capillaries. Another major compartment is the intracellular fluid (ICF), which includes the cytosol and fluid inside of cellular organelles. Of course, each of the 30–40 trillion cells in the human body, even adjacent cells, has a potentially unique set of compositions. Finally, the body surrounds many fluid compartments—Bernard’s “milieu extérieur”—that really are outside the body. These compartments include the contents of renal tubules in which urine formation occurs, alveoli in which pulmonary gas exchange occurs, and the entire gastrointestinal tract, including structures that diverge from the intestines (e.g., ducts of the pancreas). Nearly all of these countless compartments, and many of the membranes that surround them, contain CAs. Mathematical modeling holds the potential of helping us understand physiology in areas of the body where processes are currently impossible to measure or difficult to interpret. Thus, in this review, we also consider how mathematical modeling can provide insights on CA functions in tissues—that is, complex compartments—taking as specific examples renal proximal tubules as well as alveoli and their adjacent capillaries.

## 2. Role of Carbonic Anhydrases in Acid–Base Buffering

In the next sections, we review some concepts of acid–base chemistry that are important for understanding the role of CA in acid–base homeostasis. For a more in depth treatment of these topics, we refer to Ref. [50], which provides a tutorial for beginners in the field. For a more advanced treatment of these topics, we refer to Refs. [51,52].

### 2.1. Role of Buffers in Acid–Base Homeostasis

For any compartment in the body—or nanodomains adjacent to membranes or other structures—maintaining pH within a narrow range could be essential for normal physiological processes. Homeostasis is the active control—and thus the tendency to stabilize the value—of a vital physiological parameter. Integrated over many compartments, pH homeostasis is critical for life. The fine tuning of steady-state pH in a compartment is the result of adjustments in the rates at which acid–base equivalents move across the membranes bordering that compartment.

Intimately related to the concept of pH homeostasis is that of buffering. A buffer is any chemical that can reversibly consume or release H^+^:

(1) $${\mathrm{HB}}^{\left(n+1\right)}⇌B^{n}{+H}^{+}$$

In the above reaction, HB^(*n*+1)^ is a weak acid with a valence of n + 1, whereas B*^n^* is the conjugate weak base with a valence of *n*. It is important to note that buffers play no role in establishing the steady-state pH of a compartment—that depends only on the addition or consumption of acid–base equivalents. Nevertheless, buffers do play two important roles in acid–base homeostasis, but only temporary (time-dependent) ones: (1) An acute acid or alkaline load to a compartment perturbs the equilibria of a multitude of pH buffers, and the buffers reactions respond by tending to minimize the initial change in pH. (2) The compensatory response of acid–base transporters to the pH perturbation shifts these equilibria in the direction opposite to that of the original perturbation, so that the buffer reactions tend to slow the attainment of the new steady state. In other words, the role of buffers is to prevent pH from changing too far or too fast—they tend to stabilize pH [50,51,53,54].

For organisms with substantial rates of CO_2_/HCO_3_^−^ metabolism, the CO_2_/HCO_3_^−^ buffer pair—a special case of the buffer in Reaction (1)—can, in principle, be a more powerful buffer than all other buffers combined. However, this high CO_2_/HCO_3_^−^ buffering power has two requirements: (1) As we discuss below, the “system” must be “open” vis-à-vis CO_2_/HCO_3_^−^. (2) The CO_2_/HCO_3_^−^ buffer reactions must be able to progress with sufficient velocity that they can contribute in the available time—the raison d’être for carbonic anhydrases.

### 2.2. The CO_2_/HCO_3_^−^ Buffer System and Related Species

The major components of the CO_2_/HCO_3_^−^ buffer system—CO_2_, HCO_3_^−^ and H^+^—are linked through the two-step reaction

(2) $${\mathrm{CO}}_{2}+H_{2}O⇌H_{2}{\mathrm{CO}}_{3}⇌{\mathrm{HCO}}_{3}^{-}+H^{+}$$

The first reaction—the CO_2_ hydration/carbonic acid (H_2_CO_3_) dehydration reaction—is very slow. The second reaction—the dissociation or ionization of H_2_CO_3_—is extremely rapid and is always at equilibrium under physiological conditions [55]. Because many physiological processes occur in a time scale much faster than that of the CO_2_ hydration reaction, most tissues and cells in the body express a form of CA.

The reactions in (2) can be written in the thermodynamically equivalent form

(3) $${\mathrm{CO}}_{2}+H_{2}O⇌{\mathrm{HCO}}_{3}^{-}+H^{+}$$

The Henderson–Hasselbalch equation describes the equilibrium, in logarithmic form, of the above reaction and states that, in a simple CO_2_/HCO_3_^−^ buffer system, pH depends on the ratio of the concentrations of HCO_3_^−^ and dissolved CO_2_:

(4) $$\mathrm{pH}\;=\;pK_{{\mathrm{CO}}_{2}}{+\;\log}_{10}\frac{{[\mathrm{HCO}}_{3}^{-}]}{\left[{\mathrm{CO}}_{2}\right]}$$

where p*K*_CO2_ is the negative log_10_ of the equilibrium constant *K* of Reaction (3), and has a value of ~6.10 in mammalian blood plasma at 37 °C.

In addition to the above CO_2_-related reactions, several others can occur in biological solutions. For example, the HCO_3_^−^ that forms in Reaction (2) can dissociate rapidly to form carbonate (CO_3_^=^):

(5) $${\mathrm{HCO}}_{3}^{-}⇌{\mathrm{CO}}_{3}^{=}{+H}^{+}$$

This reaction, governed by a p*K* of ~10.3 at 37 °C, is of limited significance for buffering at the pH of mammalian blood plasma, but could be important for compartments at high pH. Moreover, CO_3_^=^, in turn, can form ion pairs with Na^+^, Li^+^, Ca^2+^ and Mg^2+^. Finally, Reaction (5) is crucial for certain so-called HCO_3_^−^ transporters that in fact appear to carry CO_3_^=^ [56].

Another reaction that involves CO_2_ is the direct combination with OH^−^:

(6) $${\mathrm{CO}}_{2}^{}{+\mathrm{OH}}^{-}⇌{\mathrm{HCO}}_{3}^{-}$$

Like Reaction (5), Reaction (6) is of little significance for CO_2_/HCO_3_^−^ buffering except in high-pH compartments, where [OH^−^] is high. Although the literature often simplifies the situation by stating that CAs catalyze the first step in Reaction (2), the α-CAs at the level of the catalytic zinc atom (i.e., Zn^2+^) catalyze Reaction (6), while at the same time splitting H_2_O (H_2_O ⇌ H^+^ + OH^−^) to provide the needed OH^−^. Recently, Zhao and colleagues developed a novel assay for measuring CA activity, based on stopped-flow techniques and the creation of a well-defined out-of-equilibrium condition [57].

From the above discussion, we can conclude that most CO_2_-related carbon in the human body—aside from the carbonates in structures such as bone and tooth enamel—is present in the form of HCO_3_^−^. In fact, for a pH of 7.40—the value of normal, human arterial plasma at 37 °C—the Henderson–Hasselbalch equation predicts that the concentration of HCO_3_^−^ is approximately 20 times the concentration of dissolved CO_2_. For a pH of 7.20—a representative value of normal intracellular pH of most cells—the Henderson–Hasselbalch equation predicts that the concentration of HCO_3_^−^ is approximately 12.6 times the concentration of dissolved CO_2_.

### 2.3. Competition among Buffers

H^+^ (or the OH^−^ formed in the reaction H_2_O ⇌ H^+^ + OH^−^) is the common denominator in the above reactions. In other words, these reactions are all competing for a common pool of H^+^/OH^−^. Understanding this competition is extraordinarily complex. In the 20^th^ century, physiologists quantitating acid–base chemistry typically employed the so-called Davenport diagram [58], which lumps together all non-CO_2_/HCO_3_^−^ buffers in Reaction (1) into a single pseudo-buffer, the protonation of which varies approximately linearly with pH, and ignores all reactions involving CO_3_^=^. Moreover, the Davenport approach applies only to equilibrium conditions in a single compartment. Reference [51] provides a theoretical explanation for constructing the Davenport diagram and using it to interpret acid–base disturbances. Nevertheless, the Davenport approach was a major step forward in understanding the competition among buffer reactions.

Modern computational approaches make it possible to model explicitly each individual buffer pair, as done by Somersalo et al. [59], and to simulate how the system achieves equilibrium in a time- and space-dependent manner, taking into consideration both the reactions and diffusion of each buffer component. Moreover, preliminary reports describe extensions of such models to reactions involving CO_3_^=^ [60]. A recent modeling contribution describes the reaction and diffusion processes in a confined space near a cell membrane [61].

### 2.4. CO_2_/HCO_3_^−^ Buffering in Closed vs. Open Systems

A closed system is one from which none of the components of CO_2_/HCO_3_^−^—related reactions (including any carbonate-related species) can escape, nor into which none can enter. Here, the buffering power (β_closed_)—a measure of pH stability—is maximal when pH = p*K*_CO2_ and falls off symmetrically at higher/lower pH values. In general, for a buffer pair of the form HB/B^−^, β_closed_ is

(7) $$\beta_{\mathrm{closed}}\;=\;2.3\;[TB]\frac{[H^{+}]\cdot K}{{\left([H^{+}]+K\right)}^{2}}$$

According to Equation (7), β_closed_ is proportional to the total amount of buffer, [TB]—which is the sum of [HB] and [B^−^] [51].

In a closed system, the buffering power of CO_2_/HCO_3_^−^ is relatively low, as for any other buffer pair (e.g., H_2_PO_4_^−^/HPO_4_^=^). In such a closed system, the buffering of added H^+^ in Reaction (2) is limited by the depletion of HCO_3_^−^ and build-up of CO_2_. Conversely, buffering due to H^+^ depletion is limited by the depletion of CO_2_ and build-up of HCO_3_^−^.

Although CO_2_/HCO_3_^−^ is a mediocre buffer in a closed system, it can be extremely powerful if the “system” is open to CO_2_, as is generally the case. For example, the CO_2_ in arterial blood plasma is virtually in equilibrium with the CO_2_ in alveolar air, which acts as an infinite sink for CO_2_ because of the body’s ability to regulate ventilation and thereby stabilize alveolar [CO_2_] despite large changes in the metabolic production of CO_2_. Thus, the buffering of H^+^ in Reaction (2) is limited only by the depletion or accumulation of HCO_3_^−^ because the CO_2_ produced ultimately ends up in the atmosphere. In such an open system, β_open_ for CO_2_/HCO_3_^−^ rises exponentially with pH, as follows:

(8) $$\beta_{\mathrm{open}}\;=\;2.3\;[{\mathrm{HCO}}_{3}^{-}]\;=\;2.3\;[{\mathrm{CO}}_{2}]\;10^{\left(\mathrm{pH}-pK_{{\mathrm{CO}}_{2}}\right)}$$

and—in the physiological range—can be enormous.

Because blood plasma is in direct communication with the bulk of the ECF, and because CO_2_ in the ECF equilibrates across cell membranes [62] with the CO_2_ in the cytoplasm, the human body behaves more or less as one large open system for CO_2_. The ability of the body to exploit this large CO_2_/HCO_3_^−^ buffering power requires that the CO_2_/HCO_3_^−^ buffer reaction can take place on an appropriately rapid physiological time scale, which is the job of CAs.

## 3. Role of Carbonic Anhydrases in the Facilitated Diffusion of CO_2_ and Other Buffers

The traffic of CO_2_ into or out of a compartment depends not only on the movement of CO_2_ across the border of that compartment (e.g., the plasma membrane in the case of a cell) but also on the diffusion of CO_2_ across the adjacent unstirred layers—more accurately termed unconvected layers [59]—on both sides of the membrane (i.e., intracellular and extracellular fluids in the case of a cell). Because free diffusion of CO_2_ is often slow considering the rapid time and spatial scales of physiological processes, the CO_2_/HCO_3_^−^ buffer—in addition to its role in buffering—can play a key role in facilitating (or, equivalently, augmenting) the diffusion of CO_2_ within or across the many compartments of the body.

In the next sections, we discuss how CO_2_/HCO_3_^−^-related reactions and CA can augment the diffusion of CO_2_ within aqueous unconvected layers (ULs) and across membranes. In particular, we focus our attention on some of the work done in the past 50 years and that has set important milestones towards our understanding of the contribution of the reaction and diffusion processes involved in facilitating CO_2_ diffusion.

### 3.1. Role of CA in the Facilitated Diffusion of CO_2_

“Facilitated CO_2_ diffusion” describes the contribution of HCO_3_^−^, H^+^, and other buffer pairs (HB/B^−^) to CO_2_ transport by simultaneous reaction and diffusion processes in which the parallel diffusion of HCO_3_^−^, H^+^, and HB—and the antiparallel movement of B^−^—enhances the “free” diffusion of CO_2_. As we show below, similar processes also can facilitate the diffusion of HCO_3_^−^, H^+^, B^−^, and HB.

**Figure 1:**
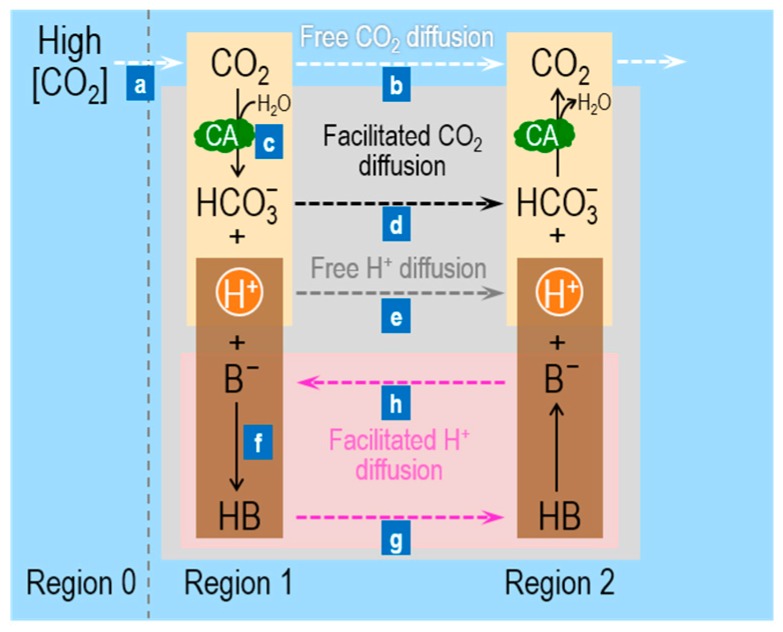
Schematic of the reaction and diffusion processes involved in facilitated CO_2_ diffusion. Before time zero, we assume that Regions 0, 1, and 2 have identical compositions, with all reactions being in equilibrium and no concentration gradients existing between Regions. At time zero, we establish a CO_2_ gradient from Region 0 (high [CO_2_]) to Region 1 (low [CO_2_]) in the presence of HB/B^−^, a generic non-CO_2_/HCO_3_^−^ buffer. Although CO_2_ is the only species moving from Region 0 to Region 1, all species can move between Region 1 and Region 2. Some CO_2_ moves from Region 1 to Region 2 by free diffusion. The shaded grey area identifies facilitated CO_2_ diffusion (i.e., HCO_3_^−^ and H^+^ moving in the same direction as CO_2_) from Region 1 to Region 2. The pink shaded area identifies facilitated H^+^ diffusion (i.e., the antiparallel movements of HB and B^−^, with HB [the weak acid] moving in the same direction as CO_2_ [the potential weak acid]). The solid arrows identify reactions, and the dashed arrows identify free solute diffusion. These reaction and diffusion events greatly accelerate the transfer of carbon (in the guise of CO_2_ or HCO_3_^−^) from Region 1 to Region 2. For details, see text. CA, carbonic anhydrase.

Figure 1 summarizes the processes (labeled “a”, “b”, “c”, etc.) involved in facilitated CO_2_ diffusion in a layer of buffered solutions (shaded grey area between Regions 1 and 2). Imagine that we begin with a layer of buffered solution that contains the CO_2_/HCO_3_^−^ buffer, CA, and a mobile buffer HB/B^−^—in equilibrium both with respect to chemical reactions and diffusion. We now suddenly raise [CO_2_] in Region 0 (to the left of Region 1 in Figure 1), thereby establishing a gradient for CO_2_ from Region 0 (R0) to Region 1 (R1)—that is, [CO_2_]_R0_ > [CO_2_]_R1_—and causing a flux of CO_2_ (“a” Figure 1). The CO_2_ newly arriving in Region 1 can have two fates (“b” and “c” in Figure 1): (b) because [CO_2_]_R1_ now exceeds [CO_2_]_R2_, CO_2_ can freely diffuse from Region 1 → Region 2 (R2, dashed white arrow); or (c) it can combine with H_2_O to form equal amounts of HCO_3_^−^ and H^+^, thereby making [HCO_3_^−^]_R1_ > [HCO_3_^−^]_R2_ and [H^+^]_R1_ > [H^+^]_R2_. In Region 1, the presence of CA promotes facilitated diffusion by two mechanisms. First, the CA promotes the consumption of CO_2_ in Region 1, and thereby magnifies the concentration gradient driving CO_2_ diffusion from Region 0 → Region 1 (“a” in Figure 1). This is the critical first step of facilitated diffusion: because of CA, carbon (in the form of CO_2_) moves more rapidly from Region 0 to Region 1. Second, CA in Region 1 promotes the transformation of most of this carbon (under physiological conditions) to HCO_3_^−^, which then diffuses from Region 1 to Region 2 (dashed black arrow; “d” in Figure 1).

The entire process outlined above ought to be pH sensitive. Under physiological conditions (e.g., pH = 7.4, p*K*_CO2_ = 6.1), the ratio [HCO_3_^−^]/[CO_2_] is 10^(7.4–6.1)^ = 20. Thus, if 21 molecules of CO_2_ diffused from Region 0 to Region 1, one would remain CO_2_ but 20 would become HCO_3_^−^ (if we assume that pH does not change). Thus, the CA-catalyzed reaction consumes ~95% of the CO_2_ arriving in Region 1, thereby enhancing the CO_2_ gradient from Region 0 → Region 1. Moreover, by converting ~95% of the incoming CO_2_ to HCO_3_^−^, the reaction increases the concentration gradient from Region 1 → Region 2 by 20× more for HCO_3_^−^ than for CO_2_. Because CO_2_ and HCO_3_^−^ have similar diffusion coefficients, the CA-enhanced conversion from CO_2_ to HCO_3_^−^ in Region 1 greatly accelerates the flux of carbon, mostly in the guise of HCO_3_^−^.

If the ambient pH were the same as the p*K*_CO2_, the contribution of facilitated diffusion would be much less. In this case, if two molecules of CO_2_ diffused from Region 0 to Region 1, one would remain CO_2_ and one would become HCO_3_^−^. Compared to the example at pH 7.4, the enhancements of both the CO_2_ gradient from Region 0 → Region 1, and of the HCO_3_^−^ gradient from Region 1 → Region 2 would be diminished.

Once the CO_2_ and HCO_3_^−^ arrive in Region 2, some of the newly arriving HCO_3_^−^ will—under the influence of CA—combine with H^+^ to form CO_2_ and H_2_O. Region 2 now becomes the new Region 1, and the facilitation process recapitulates itself indefinitely.

We now turn our attention to the non-CO_2_/HCO_3_^−^ buffer. The newly formed H^+^ in Region 1 can (see “e” and “f” in Figure 1): (e) diffuse freely from Region 1 to Region 2 (dashed grey arrow); or (f) react with B^−^ to form HB. Process “f” is important for two reasons. First, it promotes the conversion of a greater fraction of incoming CO_2_ to HCO_3_^−^. Second, because concentration gradients for H^+^ are seldom large, process “e*”* is usually very slow. On the other hand, the vast majority of newly formed H^+^ will react with B^−^ to form HB, so that the increase in [HB]_R1_ may—depending on pH, p*K*_HB_, and [TB]_R1_—be orders of magnitude greater than the increase in [H^+^]_R1_. Thus, the gradient [HB]_R1_ > [HB]_R2_ may increase by orders of magnitude more than the gradient [H^+^]_R1_ > [H^+^]_R2_, and thus the presence of the mobile buffer facilitates diffusion of H^+^ ions from Region 1 to Region 2 (pink shaded area; “g” in Figure 1). After HB arrives in Region 2, it rapidly dissociates into B^−^ and H^+^. B^−^ diffuses back to Region 1 (“h” in Figure 1), whereas the newly formed H^+^ rapidly reacts with the HCO_3_^−^ ions that have diffused from Region 1 and form CO_2_.

In summary, facilitated CO_2_ diffusion depends on four factors, aside from the initial increment in [CO_2_]_o_. Viewed from the perspective of Region 1, these are: (i) the difference (pH – p*K*_CO2_), which establishes the fraction of CO_2_ (arriving from Region 0) consumed to form HCO_3_^−^; (ii) CA, which accelerates this consumption of incoming CO_2_; (iii) the total concentration of the mobile buffer, [TB], which influences the velocity of H^+^ consumption and the magnitudes of the gradients for B^−^ and HB; and (iv) the difference (pH – p*K*_HB_), which influences the same parameters as (iii). The same four factors play a converse role as viewed from Region 2, and the eight factors together determine the fluxes through the five dashed arrows in Figure 1. Note that all solutes diffuse in the direction of the CO_2_ gradient, except B^−^, which diffuses in the direction opposite to that of HB.

The role of CA in facilitating diffusion of CO_2_ was first demonstrated in 1966 in the laboratory of Forster [63], where Longmuir and colleagues measured the apparent diffusion coefficient of CO_2_ (*D*_CO2,app_) in buffered solutions. They found that adding to the buffer solution a tiny amount of purified CA, or the CA in hemolyzed whole human blood, increased the value of *D*_CO2,app_ from that in water. These authors concluded that, in the absence of CA, the CO_2_ hydration/dehydration reactions are the rate limiting steps and that CA augments the diffusion of CO_2_. In the same year, Enns et al. [64] and Moll and Gros [65] demonstrated that facilitated diffusion of CO_2_ occurs in layers of red blood cells [64,65] and that acetazolamide (ACZ) blocks this facilitation [64]. In 1967, Enns showed that this facilitation (vs. the free diffusion of CO_2_) increases with increasing pH and becomes dominant at pH values greater than the p*K*_CO2_ of 6.10, when the concentration of HCO_3_^−^ exceeds the concentration of CO_2_ [66].

Gros and Moll [67] and Gros et al. [68] underscored the importance of facilitated H^+^ diffusion in the facilitation of CO_2_ diffusion. These authors showed that, in addition to CA, facilitated CO_2_ diffusion requires a sufficient amount of mobile buffers to provide an equivalent flux of H^+^ (i.e., HB plus H^+^ per se) equal to the flux of HCO_3_^−^—consistent with the 1-to-1 stoichiometry of H^+^ and HCO_3_^−^ in Reaction (3). They reached this conclusion by complementing their experiments with quantitative considerations [67] and mathematical modeling [68]. In their quantitative approaches, based on Fick’s first law of diffusion or on the Nernst–Planck equation in one dimension, these authors assumed that, in the presence of CA, Reaction (3) is in equilibrium and that facilitated diffusion is electrically silent. Thus, by everywhere imposing: (a) chemical equilibrium of Reaction (3) and (b) electroneutrality in the layer, and also applying (c) appropriate boundary conditions to mimic their experimental conditions, they solved numerically the resulting model equations to predict total fluxes of CO_2_ (defined as the sum of the fluxes of dissolved CO_2_ and of HCO_3_^−^) that would agree quantitatively with the fluxes of CO_2_ measured experimentally [68,69]. The model predictions supported the conclusion that soluble proteins (i.e., CAs) and mobile buffers can significantly increase H^+^ diffusion and CO_2_ transfer.

### 3.2. Role of CA in the Facilitated Diffusion of Solutes Other Than CO_2_

In addition to facilitating CO_2_ diffusion, CA can also promote the facilitated diffusion of HCO_3_^−^ and H^+^, as well as of B^−^ and HB.

**Figure 2:**
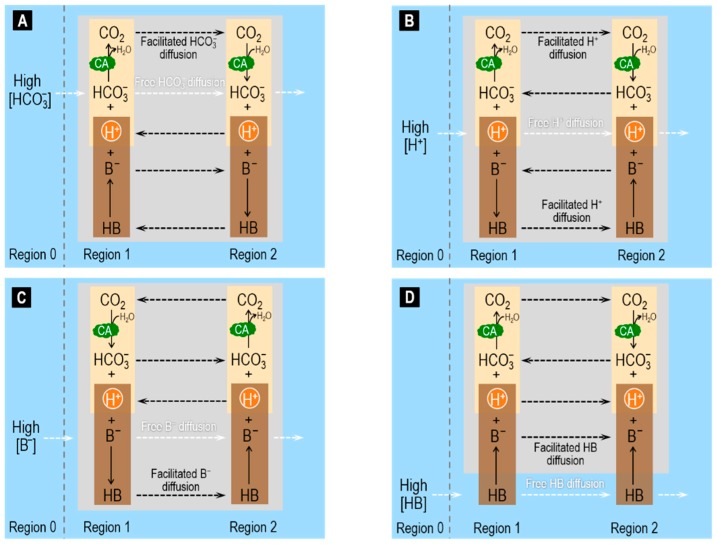
Schematic of the reaction and diffusion processes involved in facilitated diffusion of: HCO_3_^−^ (**A**); H^+^ (**B**); B^−^ (**C**); and HB (**D**). We set up the four examples as in Figure 1, except that the component that we add to Region 0 is different. At time zero, we establish a gradient for HCO_3_^−^ in (**A**) from Region 0 (high [HCO_3_^−^]) to Region 1 (low [HCO_3_^−^]), for H^+^ in (**B**), for B^−^ in (**C**), and for HB in (**D**). The solid arrows identify reactions, and the dashed arrows identify free solute diffusion. For details, see text. CA, carbonic anhydrase.

In Figure 2A, we suddenly raise only [HCO_3_^−^] in Region 0, generating a HCO_3_^−^ flux from Region 0 to Region 1. At the pH of blood (pH >> p*K*_CO2_), only a small fraction of the HCO_3_^−^ newly arriving in Region 1 goes on to form CO_2_. Thus, the diffusion of CO_2_ from Region 1 to Region 2 (i.e., the facilitated diffusion of HCO_3_^−^) augments only slightly the free HCO_3_^−^ diffusion from Region 1 to Region 2. Note that, because the non-CO_2_/HCO_3_^−^ buffer now produces H^+^ in Region 1 (rather than consuming H^+^ as in Figure 1), H^+^ now diffuses in the direction opposite that of CO_2_ and HCO_3_^−^. In summary, CO_2_ and B^−^ move in parallel with HCO_3_^−^ (the prime mover), whereas H^+^ and HB (i.e., the two acidic non-CO_2_/HCO_3_^−^ components) move antiparallel.

In Figure 2B, we suddenly raise only [H^+^] in Region 0, generating an H^+^ flux from Region 0 to Region 1. Nearly all of the H^+^ newly arriving in Region 1 goes on to form CO_2_ or HB. Thus, the diffusion of CO_2_ and HB from Region 1 to Region 2 (i.e., the facilitated diffusion of H^+^) augments greatly the free H^+^ diffusion from Region 1 to Region 2. The CA enhances the contribution of CO_2_/HCO_3_^−^ vs. HB/B^−^. In summary, H^+^ and the two acidic members of the buffer pairs (i.e., CO_2_ and HB) move in parallel with H^+^ (the prime mover), whereas the two basic members of the buffer pairs (i.e., HCO_3_^−^ and B^−^) move antiparallel. Spitzer and colleagues examined this system both in terms of physiological experiments on cardiac myocytes and modeling [70].

In Figure 2C, we suddenly raise only [B^−^] in Region 0. The system here behaves similarly to the one in which we raise only [HCO_3_^−^] (see Figure 2A) except that: (a) B^−^ replaces HCO_3_^−^; and (b) HB replaces CO_2_.

In Figure 2D, we suddenly raise only [HB] in Region 0. The system here behaves similarly to the one in which we raise only [CO_2_] (see Figure 1) except that: (a) B^−^ replaces HCO_3_^−^; and (b) HB replaces CO_2_.

### 3.3. Role of CA in the Diffusion of CO_2_ across Artificial Membranes

CA plays an important role not only in facilitating the diffusion of CO_2_ within a layer of buffer solutions but also in facilitating diffusion of CO_2_ across biological membranes. In this section, we review the reaction and diffusion processes underlying facilitated CO_2_ diffusion across membranes.

According to an integrated form of Fick’s first law of diffusion, the flux of CO_2_ across a membrane (*J*_M,CO2_) is equal to the product of the permeability of the membrane to CO_2_ (*P*_M,CO2_) times the difference in the concentration of CO_2_ across the membrane:

(9) $$J_{{M,\mathrm{CO}}_{2}}=P_{{M,\mathrm{CO}}_{2}}\cdot {([\mathrm{CO}}_{2}{]}_{\mathrm{out},\mathrm{aq}}-{{[\mathrm{CO}}_{2}]}_{\mathrm{in},\mathrm{aq}})$$

where [CO_2_]_out,aq_ is the concentration of CO_2_ in the aqueous layer immediately adjacent to the extracellular or outer surface (“out”) of the membrane and [CO_2_]_in,aq_ is the concentration of CO_2_ in the aqueous layer immediately adjacent to the intracellular or inner surface (“in”) of the membrane. With these definitions of [CO_2_], *P*_M,CO__2_ is the permeability of the membrane per se. If we instead defined the [CO_2_] values as those in the bulk solution at some distance from the membrane, then the permeability would be less because it would include the effects of the resistance to diffusion offered by the unconvected layers on either side of the membrane [62].

According to Equation (9), *J*_M,CO2_ can increase because of an increase in *P*_M,CO2_ and/or because of an increase in the transmembrane CO_2_ concentration difference. Integral membrane proteins such as aquaporins (AQPs) or Rhesus (Rh) can increase *J*_M,CO2_ by increasing *P*_M,CO2_ [59,71,72,73,74,75,76]; CAs can increase *J*_M,CO2_ by maximizing transmembrane CO_2_ concentration differences [77,78,79].

**Figure 3:**
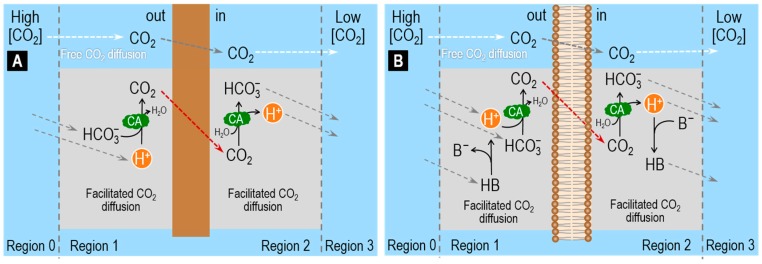
Schematic of the reaction and diffusion processes involved in facilitated CO_2_ diffusion up to and away from membranes that are permeable only to CO_2_. Before time zero, we assume that all Regions have identical compositions, with all reactions being in equilibrium and no concentration gradients existing between adjacent Regions. At time zero, we establish a CO_2_ gradient from Region 0 to Region 1. (**A**) Silicone rubber artificial membrane, with no buffers other than CO_2_/HCO_3_^−^ in the aqueous solutions (Broun model [80,81]). Here, the CA is attached to both sides of the membrane (“enzymatic coating”), but is present nowhere else in the system. In this system, a low level of facilitated CO_2_ diffusion—low because CA is not in the bulk solution and no non-CO_2_/HCO_3_^−^ buffers are present—would speed the appearance of CO_2_ on the left side of the membrane, enhancing the gradient for CO_2_ to cross the membrane. In addition, a low level of facilitated CO_2_ diffusion would speed the removal of CO_2_ on the right side of the membrane, again enhancing the transmembrane CO_2_ gradient. (**B**) Artificial planar lipid bilayer, with the HB/B^−^ buffer pair present in both aqueous solutions, and soluble CA present throughout the aqueous solutions (Gutknecht–Tosteson model [82]). Here the facilitated diffusion of CO_2_—from Region 0 to 1, within Region 1, from Region 1 to Region 2, within Region 2, and from Region 2 to Region 3—is faster than in (**A**) for two reasons: the presence of CA and the presence of HB/B^−^. Both speed CO_2_ facilitated diffusion in Regions 1 and 2. As a result, the gradient of CO_2_ across the membrane—and thus the transmembrane flux—is larger in (**B**) than in (**A**).

Several authors have investigated facilitated CO_2_ diffusion across hydrophobic membranes. For example, Broun et al. used artificial membranes, made of silicone rubber, and “enzymatic coating”, to study the mechanisms by which “interfacial enzymatic reactions”, such as CA, may facilitate diffusion of CO_2_ across a membrane [80,81]. By employing pH-stat recordings and mathematical modeling, they found that transmembrane CO_2_ fluxes are doubled when CA is on the surface of the membrane [81]. These authors employed a steady-state one-dimensional reaction–diffusion model to predict the concentration profiles of CO_2_ and HCO_3_^−^ in the boundary layers of unconvected aqueous solution adjacent to the membrane, when CO_2_ permeates the membrane (Figure 3A). The model predicts that CA, by increasing the rate of Reaction (3), causes steeper HCO_3_^−^ concentration gradients in the ULs adjacent to the hydrophobic membrane and also steeper CO_2_ concentration gradients across the membrane [80,81].

In 1977, Gutknecht et al. used ^14^C-labeled CO_2_ to measure the diffusive flux of CO_2_ across artificial planar lipid bilayers (made of cholesterol, egg lecithin and decane) and adjacent ULs [82]. They demonstrated that, in the absence of CA in the bathing solutions, Reaction (2) is too slow to allow efficient conversion of HCO_3_^−^ into CO_2_ in the UL adjacent to the outer side of the membrane and, therefore, is unable to facilitate CO_2_ diffusion. In the presence of CA in the bathing solutions, rapid conversion of HCO_3_^−^ into CO_2_ sustains a relatively high [CO_2_] in the UL on the upstream side of the membrane thereby facilitating diffusion of CO_2_ through the artificial lipid bilayer membrane. These authors emphasized the important role of non-CO_2_/HCO_3_^−^ buffers (i.e., HB/B^−^) in facilitating CO_2_ diffusion across a membrane (Figure 3B). Indeed, they observed that CA facilitates CO_2_ diffusion only when a mobile non-CO_2_/HCO_3_^−^ buffer (such as Tris or HEPES) is also present in the ULs. As illustrated in Figure 3B, the non-CO_2_/HCO_3_^−^ buffer supplies the H^+^ needed to combine with HCO_3_^−^ to produce CO_2_ in the UL adjacent to the outer side of the membrane (out) and removes the H^+^ produced by the CO_2_ hydration reaction in the UL adjacent to the inner side (in) of the membrane [82,83].

In their study, Gutknecht et al. combined biophysical experiments with a theoretical analysis of the diffusion of CO_2_ through ULs surrounding a bilayer membrane. The bilayer separates two regions with identical bathing solutions except for the presence of tracer (i.e., ^14^C) in Region 1. By assuming that

1. CO_2_, HCO_3_^−^ and CO_3_^=^ are in chemical equilibrium, that is, the reactions among these solutes occur very rapidly (i.e., CA is present), at a rate much faster than the rate of solute diffusion across the ULs and the membrane;
2. only HCO_3_^−^ and CO_3_^=^ carry the tracer and diffuse through the ULs; and
3. only CO_2_ moves across the membrane,

These authors were able to use a simplified form of an equation developed earlier [83,84] to relate the steady-state one directional diffusive flux of total CO_2_ (*J*_CO2_) to the diffusive flux of A^−^ in the UL and the transmembrane flux of CO_2_:

(10) $$\frac{1}{J_{{\mathrm{CO}}_{2}}}=\frac{1}{P_{\mathrm{UL},A^{{}^{-}}}\cdot {[A}^{-}]}+\frac{1}{P_{{M,\mathrm{CO}}_{2}}\cdot {[\mathrm{CO}}_{2}]}$$

where *P*_UL,B_−__ is the permeability of the UL to A^−^, [A^−^] is the sum of the concentrations of HCO_3_^−^ and CO_3_^=^ in Region 1 (the region where the flux of CO_2_ originates) and *P*_M,CO2_ has the usual meaning.

Gutknecht et al. employed the above equation to calculate *J*_CO2_ as a function of [A^−^], at a constant [CO_2_] (i.e., as the pH increases). Moreover, by taking advantage of the catalytic action of CA and by using different pH ranges, these authors were able to identify a condition (CA in the bath and pH > 9) that allowed them to estimate *P*_M,CO2_ by fitting the above equation to their experimental data. In this case, *J*_CO2_ saturates because the ratio of [A^−^]/[CO_2_] is so high that diffusion of A^−^ in the UL dominates diffusion of CO_2_ across the membrane.

### 3.4. Role of CA in the Diffusion of CO_2_ across the Membrane of a Living Cell

A recent series of three companion papers from our group examined the role of CAs (cytosolic CA II or extracellular-surface CA IV) and non-CO_2_/HCO_3_^−^ buffers on the fluxes of CO_2_ across the membrane of a living cell—a *Xenopus laevis* oocyte [77,78,79]. For this type of study, oocytes are useful model systems because of their negligible native membrane permeability to H^+^ and HCO_3_^−^-related species.

**Figure 4:**
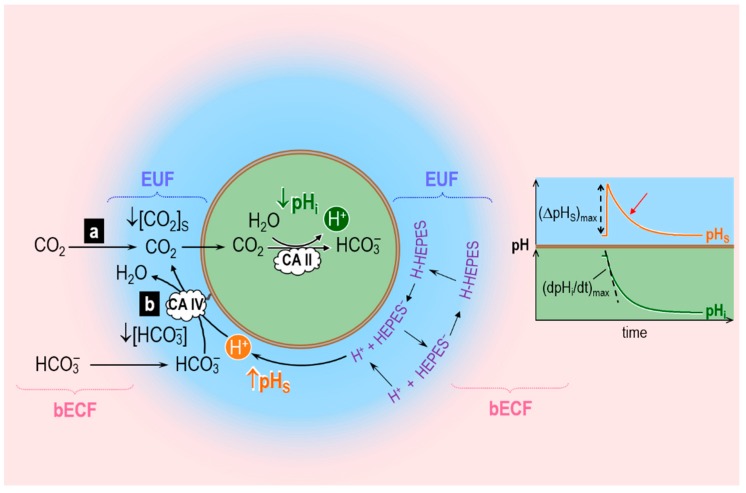
Reaction and diffusion events caused by influx of CO_2_ into a cell (an oocyte) and effect on extracellular-surface pH (pH_S_) and intracellular pH (pH_i_). Here, the influx of CO_2_ into the cell creates a CO_2_ sink at the outer surface of the membrane, ↓ [CO_2_]_S_. The two insets show the time courses of pH_S_ and pH_i_ during the CO_2_ influx. bECF, bulk extracellular fluid; EUF, extracellular unconvected fluid; CA, carbonic anhydrase. Modified from Figure 1 of [78].

Musa-Aziz et al. assessed transmembrane CO_2_ fluxes by using liquid-membrane pH-sensitive microelectrodes to measure simultaneously the changes in intracellular pH (pH_i_) and extracellular-surface pH (pH_S_) caused by the addition (or removal) of equilibrated CO_2_/HCO_3_^−^ solution in (or from) the bulk extracellular fluid (bECF). Figure 4 illustrates the principles behind the pH_i_ and pH_S_ changes caused by CO_2_ addition when CO_2_ is the only solute that can permeate the plasma membrane. The opposite occurs in the case of CO_2_ removal. Addition of equilibrated CO_2_/HCO_3_^−^ in the bECF causes [CO_2_] to rise in the unconvected fluid in contact with the cell membrane, leading to an influx of CO_2_ into the cell. Because of this CO_2_ influx, [CO_2_] at the outer surface of the cell ([CO_2_]_S_) never reaches [CO_2_]_bECF_. This cell-surface CO_2_ lost to the interior of the cell can be replenished by two processes: (a) diffusion of CO_2_ from the bECF; and (b) the reaction HCO_3_^−^ + H^+^ → CO_2_ + H_2_O at the cell outer surface. Process (a) does not involve any changes in [H^+^], and therefore does not register measurements via a pH microelectrode. Process (b) involves the consumption of H^+^ and therefore can be measured by a pH microelectrode. Musa-Aziz et al. used a blunt pH-sensitive microelectrode, gently pushed against the oocyte surface, to measure the rapid pH_S_ increase caused by process (b) (see Figure 4, orange trajectory) followed by the pH_S_ decay that parallels the decay in the CO_2_ influx as CO_2_ levels inside the cell reach those on the outside. In the cytosol, the entry of CO_2_ causes pH_i_ to decay (Figure 4, dark green trajectory) because of the intracellular reaction CO_2_ + H_2_O → HCO_3_^−^ + H^+^.

In their experiments with oocytes injected with CA II, Musa-Aziz et al. found that cytosolic CA II not only increases the maximal rate of intracellular acidification, (dpH_i_/dt)_max_—as one would expect because CA II accelerates the intracellular reaction CO_2_ + H_2_O → HCO_3_^−^ + H^+^—but also the maximal change of the pH_S_ transient, (ΔpH_S_)_max_. The cytosolic CA also shortens the time constant (τ) as pH_S_ decays from its peak (red arrow in Figure 4)—another index of an enhanced CO_2_ influx. (That is, the same net amount of CO_2_ enters the cell, but over a shorter time.) Pretreatment of CAII-injected oocytes with ethoxzolamide (EZA, a permeant CA II inhibitor) completely reversed the effects of CA II on the pH_i_ and pH_S_ transients. Thus, these authors hypothesized that cytosolic CA II, by accelerating the intracellular reaction CO_2_ + H_2_O → HCO_3_^−^ + H^+^, maintains a relatively low [CO_2_]_i_ early during the CO_2_ exposure, thereby maximizing the transmembrane gradient of CO_2_ that drives CO_2_ influx. This greater influx of CO_2_ early during the CO_2_ exposure enhances the decrease of [CO_2_]_S_, thereby accentuating (ΔpH_S_)_max_. These experiments with CA II lead to an important principle: the most straightforward pH-related approach for assessing the effects of a cytosolic CA on CO_2_ fluxes is to measure pH_S_, that is, the pH transient on the side of the membrane “trans” to the CA.

Musa-Aziz et al. also examined the effect of CA IV, which is present at the outer cell surface of the cell (Figure 4). They concluded that, by accelerating the reaction HCO_3_^−^ + H^+^ → CO_2_ + H_2_O at the outer cell surface, extracellular-surface CA IV maintains a relatively high [CO_2_]_S_, thereby maximizing the transmembrane gradient of CO_2_ that drives CO_2_ influx. These experiments with CA IV also lead to an important principle: the most straightforward pH-related approach for assessing the effects of a cell-surface CA on CO_2_ fluxes is to measure (dpH_i_/dt)_max_, that is, the pH transient on the side of the membrane “trans” to the CA.

**Figure 5:**
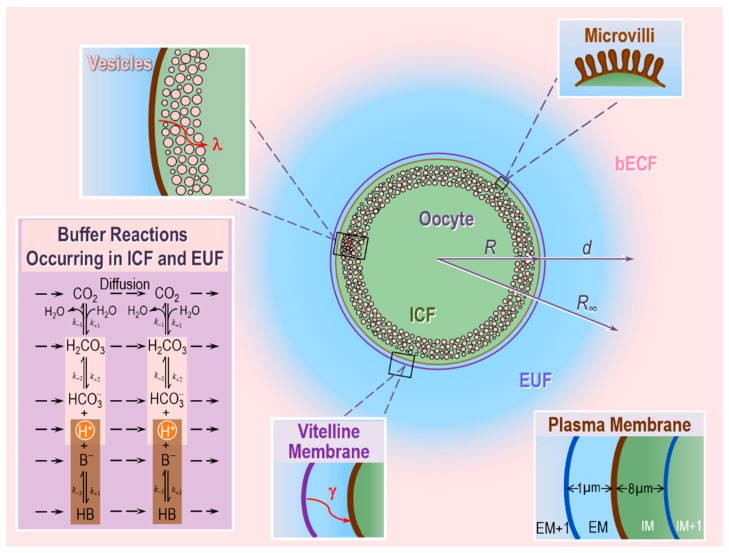
Key elements of the reaction–diffusion mathematical model of CO_2_ fluxes across the plasma membrane of an oocyte. The model includes: a thin layer of vesicles beneath the plasma membrane (inset in top left corner), folds and microvilli at the plasma membrane (PM; inset in top right corner), intracellular and extracellular buffer reactions (inset in bottom left corner), cytosolic water volume that is lower than total intracellular volume, lower diffusion constant for solutes in cytosol vs. extracellular fluid, and the vitelline membrane that envelops the PM (inset in bottom center). The inset in the bottom right corner illustrates the spatial discretization in the proximity of the PM. For additional details, see text. bECF, bulk extracellular fluid; EUF, extracellular unconvected fluid; ICF, intracellular fluid; EM, region immediately adjacent to the extracellular side of the plasma membrane; IM, region immediately adjacent to the intracellular side of the plasma membrane; *R*, radius of oocyte; *R*_∞_, radius of the computational domain; *d*, thickness of the EUF; λ and γ, tortuosity factors; *k*, reaction rate. Modified from Figure 1 of [79].

To test the above hypotheses on the role of CA II and CA IV in enhancing CO_2_ fluxes, these authors extended the earlier reaction–diffusion model developed by Somersalo et al. to describe the influx of CO_2_ influx into an oocyte [59]. Following Somersalo et al., Occhipinti et al. assumed that the system contains three major regions (see Figure 5):

1. The oocyte, a sphere that comprises the cell membrane and intracellular fluid. Within the ICF, reactions among buffers and diffusion of solutes occur. Surrounding the oocyte is…
2. The extracellular unconvected fluid (EUF), a spherical annulus that is concentric with the oocyte. Within the EUF, reactions among buffers and diffusion of solutes occur. Conceptually, the EUF is similar to the classical unstirred layer (a steady-state concept, with a characteristic value for each solute), except that the EUF has meaning even before the establishment of a steady state, and the EUF has the same thickness for each solute [59]. Surrounding the EUF is…
3. The bECF, an infinite reservoir of pre-equilibrated solution that mimics the composition of the bath solution used in the physiological experiments. No reaction or diffusion occurs in the bECF.

The ICF and EUF communicate through the plasma membrane, which is infinitely thin and permeable only to CO_2_, which can freely diffuse across the membrane according to Fick’s first law of diffusion. The EUF and bECF communicate via diffusion only. The buffer reactions in the ICF and EUF include those of: (a) the CO_2_/HCO_3_^−^ buffer, modeled according to the two-step Reaction (2), and (b) a single non-CO_2_/HCO_3_^−^ buffer of the form HB ⇌ B^−^ + H^+^. In the ICF, the HB/B^−^ buffer mimics the sum of all intrinsic buffers (i.e., the buffers whose components do not cross the plasma membrane) that are present in the ICF of an oocyte. In the EUF, the HB/B^−^ buffer represents the mobile HEPES buffer used in typical physiological experiments. The catalytic activity of CA is implemented by multiplying the rate constants of the first step of Reaction (2) by the same acceleration factor A. Specifically, the extracellular-surface CA-like activity, which mimics expression of CA IV on the outer surface of the oocyte, is implemented by multiplying the rate constants of the first step of Reaction (2) by the same factor A_S_ (acceleration factor for surface CA-activity) only in the region immediately adjacent to the extracellular surface of the plasma membrane (EM, see Figure 5, inset at the bottom right). The intracellular/cytosolic CA-like activity, which mimics CA II, is implemented by multiplying the rate constants of the first step of Reaction (2) by the same factor A_i_ (acceleration factor for intracellular CA-activity) everywhere inside the cell.

Assuming spherical radial symmetry, the concentration *C* of each solute *s*, *C_s_*, changes in time and space (radial distance *r* from the center of the cell) according to the reaction–diffusion equation

(11) $$\frac{\partial }{\partial t}C_{s}(t,r)=\underset{\mathrm{Diffusion}\;\mathrm{term}}{\underset{︸}{\frac{1}{r^{2}}\frac{\partial }{\partial r}\left(D_{s}(r)\;r^{2}\frac{\partial }{\partial r}C_{s}(t,r)\right)}}+\underset{\mathrm{Reaction}\;\mathrm{term}}{\underset{︸}{\sum_{\ell =-3,\ell \neq 0}^{+3}S_{s,\ell }\Phi_{\ell }}}.$$

Note that *D_s_* is the diffusion coefficient of solute *s*, which can change in space, *S_s,__l_* are the stoichiometry coefficients and Φ_l_ are the reaction fluxes.

The resulting system of coupled partial differential equations (PDEs), with appropriate boundary and initial conditions, is solved in MATLAB using the numerical implementation proposed in [59].

To simulate the physiological experiments of the two papers by Musa-Aziz et al. [77,78], Occhipinti et al. in their companion paper optimized the theoretical model of Somersalo et al. [59] by introducing new features, including: the vitelline membrane surrounding the oocyte, a surface amplification factor to account for folds and microvilli, a layer of intracellular vesicles beneath the plasma membrane, reduced cytosolic water content, reduced cytosolic diffusion of solutes, and a new protocol for simulating delivery and removal of the bulk extracellular CO_2_/HCO_3_^−^ solution [79]. This more refined and realistic model reproduces the essential features of the measured pH_i_ and pH_S_ transients for experiments with control, CA II-injected, and CA IV-expressing oocytes, under various experimental conditions (e.g., different extracellular CO_2_/HCO_3_^−^ levels, different extracellular HEPES levels).

Using the solute concentrations predicted by the model as a function of time and space, the authors calculated the transmembrane fluxes of CO_2_ as well as the diffusive and reaction fluxes of all solutes near the outer and inner side of the membrane. The model confirmed the hypothesis that CA IV and CA II markedly accelerate transmembrane CO_2_ fluxes by replenishing CO_2_ on the side of the membrane from which CO_2_ departs and consuming CO_2_ on the side to which CO_2_ goes.

**Figure 6:**
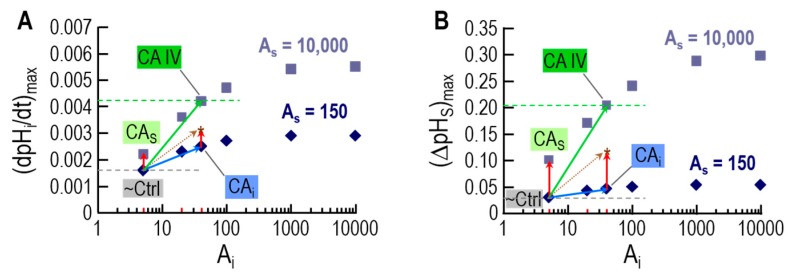
Supra-additive effects of intracellular and extracellular-surface CA activities on kinetics of pH changes, as predicted by mathematical modeling. The insets to Figure 4 show the protocol of the physiological experiment: the addition of CO_2_/HCO_3_^−^ to the extracellular fluid causes: (a) a quasi-exponential decrease of intracellular pH (pH_i_); and (b) a rapid increase in the pH at the extracellular surface (pH_S_), followed by a quasi-exponential decay. (**A**) The maximal rate of the pH_i_ decrease—(dpH_i_/dt)_max_—for 12 simulations, 6 varying the intracellular CA acceleration factor (A_i_) at a fixed extracellular-surface CA acceleration factor (A_S_) of 150 (diamonds), and 6 varying A_i_ at a fixed A_S_ of 10,000 (squares). (**B**) The maximal shift in pH_S_—(ΔpH_S_)_max_—for the same 12 simulations in (**A**). Each of the 12 simulations models a hypothetical oocyte exposed to a bulk solution containing 1.5% CO_2_/10 mM HCO_3_^−^ at pH 7.50. (**A**) Dependence of (dpH_i_/dt)_max_ on A_i_, for each of the two different values of A_S_. (**B**) Dependence of (ΔpH_S_)_max_ on A_i_, for each of the two different values of A_S_. In each panel, the six diamonds represent an isopleth for A_S_ = 150, whereas the six squares represent an isopleth for A_S_ = 10,000. In each panel, the diamond labeled “~Ctrl” (i.e., A_i_ = 5, A_S_ = 150) represents a “control” oocyte that is, an oocyte not expressing or injected with any heterologous protein. For “~Ctrl”, the (dpH_i_/dt)_max_ value in (**A**) and the (ΔpH_S_)_max_ value in (**B**) approximately match the mean data for physiological experiments, indicated by the horizontal dashed gray lines. In each panel, the square labeled “CA IV” (i.e., A_i_ = 40, A_S_ = 10,000) represents an oocyte expressing CA IV, with (dpH_i_/dt)_max_ and (ΔpH_S_)_max_ values approximately matching the mean data for physiological experiments, indicated by the horizontal dashed green lines. In each panel, the square labeled “CA_S_” (i.e., A_i_ = 5, A_S_ = 10,000) represents a hypothetical oocyte that—compared to “~Ctrl”—has an isolated increase in A_S_. Note that the (dpH_i_/dt)_max_ and (ΔpH_S_)_max_ values for this hypothetical “CA_S_” increase by amounts (red vector) that are far smaller than those actually observed for real oocytes expressing CA IV (i.e., vertical distances between horizontal dashed green and grey lines). The only way for the simulations to explain the physiological data is if CA IV expression not only raises A_S_ from 150 to 10,000 but also raises A_i_ from 5 to 40 (green vector). Indeed, physiological evidence indicates that A_i_ must indeed increase. In each panel, the diamond labeled “CA_i_” (i.e., A_i_ = 40, A_S_ = 150) represents a hypothetical oocyte with a cytosolic CA activity that is the same as that postulated for an oocyte actually expressing CA IV (blue vector). The sum of the red vector (increase A_S_ only) and the blue vector (increase A_i_ only) is the point labeled “*” (dashed golden vector). Notice that the green vector predicts that CA IV expression (combined increases in A_S_ and A_i_) produces much larger increases in (dpH_i_/dt)_max_ and (ΔpH_S_)_max_ than the dashed golden vector (the sum of isolated increases in A_S_ and A_i_). In other words, the effects of simultaneously increasing A_S_ and A_i_ are supra-additive. Modified from Figure 13 in [79].

An important insight is that—for experiments with oocytes expressing CA IV—the model can reproduce pH_i_ and pH_S_ transients from physiological experiments only if a small additional amount of CA-like activity is also implemented in the cytosol. This finding was in line with the experiments with inhibitors in oocytes expressing CA IV, and helped in reaching the conclusion that expression of CA IV in oocytes leads to the appearance of a low concentration of CA in a cytosol-accessible compartment. Figure 6 illustrates the results of the simulations that lead to this conclusion.

As shown by the diamonds in Figure 6A,B, increasing intracellular CA activity (*x*-axis) even to very high levels (i.e,. A_i_ = 5 at point labeled “~Ctrl” → A_i_ = 10,000 at extreme right) has relatively little effect on either (dpH_i_/dt)_max_ or (ΔpH_S_)_max_ when extracellular-surface CA activity is relatively low (A_S_ = 150). Conversely, a very large increase in extracellular-surface CA activity (i.e., A_S_ = 150 at point labeled “~Ctrl” → A_S_ = 10,000 at point labeled “CA_S_”) has relatively little effect on either (dpH_i_/dt)_max_ or (ΔpH_S_)_max_ when intracellular CA activity is low (A_i_ = 5). However, the combination of increasing levels of CA activity in both locations produces much larger increases in both (dpH_i_/dt)_max_ and (ΔpH_S_)_max_. For example, the point labeled “CAIV” mimics oocytes expressing CA IV, and supports the conclusion (previous paragraph) that expression of CA IV leads to increases in CA activity not only on extracellular-surface, but also in the cytosol.

The novel prediction from the simulation data in Figure 6 is that the effects of a cytosolic and extracellular-surface CA activity are supra-additive with respect to (dpH_i_/dt)_max_ and (ΔpH_S_)_max_, and thus CO_2_ diffusion across the membrane.

## 4. Role of Carbonic Anhydrases in Whole-Body Acid–Base Homeostasis: Transport of HCO_3_^−^ and CO_2_

A vital parameter for mammals is the value of arterial blood pH, which the human body maintains within a narrow range around ~7.40 under normal physiological conditions [51]. In line with the Henderson–Hasselbalch equation (Equation (4)), blood pH depends on the ratio of [HCO_3_^−^] to [CO_2_]. Because the kidneys regulate plasma [HCO_3_^−^] and the lungs regulate plasma [CO_2_], the stability of blood pH depends on the dual (and independent) action of the kidneys and the lungs. Because CA catalyzes the interconversion of CO_2_ into HCO_3_^−^ through Reaction (2), this enzyme plays a major role in both the kidneys and the lungs. In the kidneys, CA is essential for HCO_3_^−^ reabsorption and H^+^ secretion. In the pulmonary and systemic capillaries, CA is essential for CO_2_ transport across membranes, and thus carriage of CO_2_ from the systemic tissues to the alveoli for elimination from the body during expiration.

In the next two subsections, we review how CA—by impacting renal and respiratory physiology—plays a central role in whole-body pH homeostasis, how the inhibition of CA impacts acid–base balance, and how mathematical modeling can contribute to our understanding of the underlying processes.

### 4.1. The Renal System: H^+^ Secretion and HCO_3_^−^ Reabsorption

Although CAs are widely distributed throughout the kidneys, CA II and CA IV dominate in human kidneys [41]. Cytosolic CA II accounts for ~95% of total renal CA activity and is expressed virtually everywhere along the nephron, except the thin ascending limb and the preceding turn of Henle’s loop [41]. The membrane-associated CA IV and CA XII account for the remaining 5% of total renal CA activity [41]. CA IV is expressed in the apical and basolateral membranes of both the early and middle portions of the proximal tubule (PT) as well as the entire thick ascending limb [41,85]. More distally, CA IV is expressed only on the apical membranes of the α-intercalated cells of later cortical segments as well as in the principal cells of the inner and outer medullary collecting ducts [41]. CA XII immunoreactivity is distinct in the basolateral membranes of the thick ascending limb, distal convoluted tubule, and principal cells of the initial collecting tubule and later nephron segments [86].

CAs in the kidneys play a major role in the secretion of H^+^ into the tubule lumen, followed by the movement of HCO_3_^−^ into the interstitial fluid and blood. These activities achieve the final urinary acid–base composition and help maintain whole-body acid–base homeostasis. The proximal tubule is the major site of HCO_3_^−^ reabsorption/H^+^ secretion, followed by the thick ascending limb and the distal nephron. The proximal tubule reabsorbs ~80% of the HCO_3_^−^ that the glomeruli filter, and that otherwise would be lost in the urine, causing a life-threatening metabolic acidosis [87].

**Figure 7:**
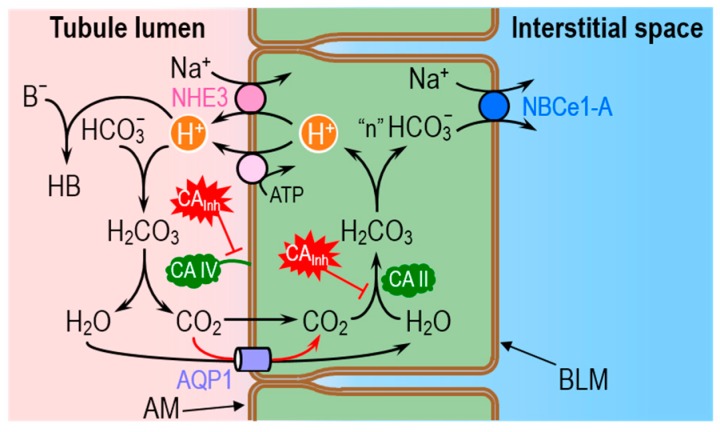
Classical model of HCO_3_^−^ reabsorption and titration of non-CO_2_/HCO_3_^−^ luminal weak bases by the renal proximal tubule (PT). According to the classical view, the only acid–base transporters at the apical membrane (i.e., the membrane facing the tubule lumen) are the Na-H exchanger NHE3 and the vacuolar-type H^+^ pump. The energy for Na-H exchange comes from the inward Na^+^ gradient, established by the Na-K pump on the basolateral membrane (not shown), and the energy for the apical H^+^ pump comes from ATP hydrolysis. HCO_3_^−^ reabsorption (or reclamation) occurs when the H^+^ secreted in the tubule lumen rapidly combines with HCO_3_^−^ (previously filtered in the glomerulus) to form H_2_CO_3_, which slowly dissociates to form H_2_O and CO_2_. Although the figure shows the GPI-linked enzyme carbonic anhydrase (CA) IV as catalyzing the slow dehydration of H_2_CO_3_, in fact CA IV bypasses this slow step by catalyzing the direct conversion of HCO_3_^−^ and H^+^ to H_2_O and CO_2_. The CO_2_ and H_2_O newly formed in the lumen then diffuse into the PT cytosol, mostly through the channel aquaporin AQP1. In the cytosol, although the figure shows cytosolic CA II catalyzing the slow hydration of CO_2_ to form H_2_CO_3_, in fact, the CA II directly converts CO_2_ and H_2_O to H^+^ and HCO_3_^−^. The H^+^ recycles back into the lumen. The HCO_3_^−^ exits the PT cell across the basolateral (i.e., blood-side) membrane via the electrogenic Na/HCO_3_ cotransporter, which exports the equivalent of 2 or 3 HCO_3_^−^ ions with 1 Na^+^ ion. The process just described merely reclaims previously filtered HCO_3_^−^; it does not titrate any acids in the body. A second fate of the H^+^ secreted in the lumen can be its combination with luminal NH_3_—which the PT cell generates from glutamine and glutamate—to form NH_4_^+^, most of which appears in the urine. This process is termed ammonium secretion. A third fate of the secreted H^+^ is to titrate weak bases other than HCO_3_^−^ or NH_3_ (e.g., phosphate, creatinine) to form the conjugate weak acid. The amount of such acid is termed the titratable acidity. In the figure, we show all of these non-HCO_3_^−^ titrations as the idealized reaction B^−^ + H+ → HB. Of course, B can have any valence (e.g., 0 in the case of NH_3_), and HB has a valence 1 greater than that of B (+1 in the case of NH_4_^+^). A fourth and final fate of the secreted H^+^ is to remain unbuffered and thereby lower the pH of the tubule fluid. Whether the secreted H^+^ titrates NH_3_ or another non-CO_2_/HCO_3_^−^ buffer, or remains unbuffered, one “new HCO_3_^−^” moves via NBCe1-A into the interstitial space to titrate acids throughout the body. AM, apical membrane; BLM, basolateral membrane; CA_Inh_, carbonic anhydrase inhibitor.

Figure 7 illustrates the basic cellular mechanisms of HCO_3_^−^ reabsorption in proximal tubule cells, and the legend provides a more detailed summary of the processes. Briefly, as the tubule cells secrete H^+^ into the lumen—via the Na-H exchanger 3 and vacuolar H^+^ pumps—a tiny fraction of the H^+^ titrates non-HCO_3_^−^ (B^−^) to form the conjugate weak acids (HB). Most of the secreted H^+^ titrates the filtered load of HCO_3_^−^ to form H_2_CO_3_, which ultimately dissociates into CO_2_ and H_2_O. The majority of the newly formed CO_2_ and H_2_O molecules move into the tubule cell via the water channel aquaporin 1 (AQP1) [71,75,88,89]. In the cytosol, CO_2_ and H_2_O rapidly recombine to form H_2_CO_3_, which then dissociates to regenerate intracellular HCO_3_^−^ and H^+^. It is the slow CO_2_ + H_2_O ⇌ H_2_CO_3_ reaction in this two-step sequence that the GPI-linked CA IV bypasses in the lumen, and that the CA II bypasses in the cytosol. The HCO_3_^−^ newly formed in the cytosol exits the tubule cell across the basolateral membrane (via the Na/HCO_3_ cotransporter NBCe1-A) into the interstitial space and ultimately in the blood. The H^+^ recycles back into the lumen.

**Figure 8:**
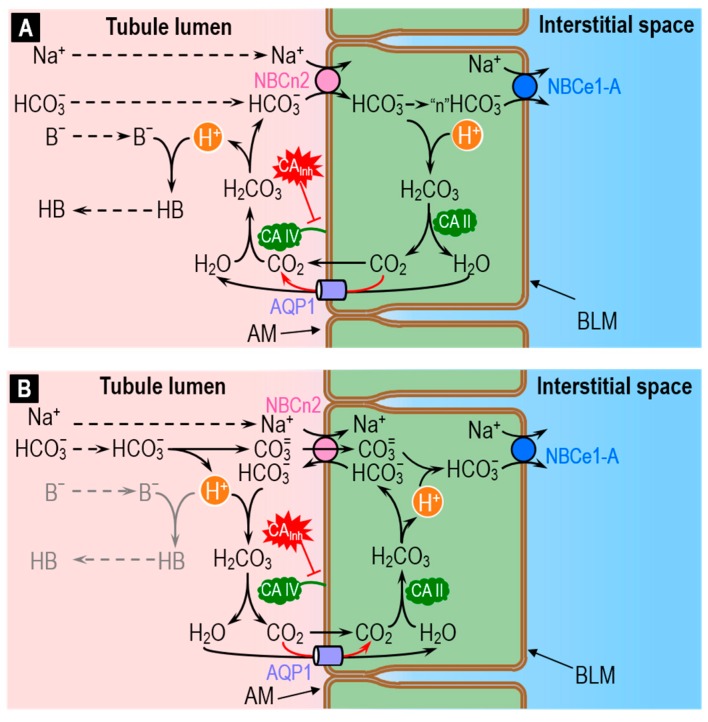
Two models of how the electroneutral Na/HCO_3_ cotransporter NBCn2 could mediate the reabsorption of HCO_3_^−^ and titration of non-CO_2_/HCO_3_^−^ buffers in the renal proximal tubule (PT). Guo et al. discovered that a variant of NBCn2 (first 4 N-terminal amino acids: MCDL) is present at the apical membrane of proximal-tubule (PT) cells [90]. Although NBCn2 is generally depicted as mediating NaHCO_3_ uptake (**A**), evidence suggests that it in fact is an exchanger. If so, the simplest model is that it exchanges Na^+^ plus CO_3_^=^ for HCO_3_^−^ (**B**). (**A**) NBCn2 as a cotransporter. If the apical NBCn2 directly moves the filtered Na^+^ and HCO_3_^−^ into the cytosol, the result would be a deficit of Na^+^ and HCO_3_^−^ in the nanodomains near the transporter. One means of replenishing the lost NaHCO_3_ would be the diffusion of Na^+^ and HCO_3_^−^ from the bulk luminal fluid to the apical membrane. Another means of replenishing the HCO_3_^−^ would be the conversion of CO_2_ and H_2_O to HCO_3_^−^ and H^+^, facilitated by apical CA IV. Our simulations [90] suggest that the reaction (H_2_O + CO_2_ → H^+^ + HCO_3_^−^) accounts for only a small fraction of total HCO_3_^−^ replenishment. The newly produced HCO_3_^−^ would move into the cytosol via NBCn2, whereas the newly produced H^+^ would either titrate B^−^ to form HB, or remain unbuffered. In the cytosol, most of the HCO_3_^−^ arriving via NBCn2 would diffuse toward the basolateral membrane, for exit via the electrogenic Na/HCO_3_ cotransporter NBCe1-A. However, a small fraction of the entering HCO_3_^−^ would in principle, under the catalytic action of CA II, recycle back into the lumen—mostly via aquaporin 1 (AQP1)—as H_2_O and CO_2_. This diffusion of H_2_O and CO_2_ is in the direction opposite shown in Figure 7, but would ultimately be responsible for the titration of B^−^ to HB. In a real PT, the H_2_O and CO_2_ necessary to replenish luminal HCO_3_^−^ could come from Na-H exchange and H^+^ pumping, as shown in Figure 7. (**B**) NBCn2 as an exchanger. In this model, filtered Na^+^ and HCO_3_^−^ diffuses from the bulk luminal fluid towards NBCn2. The HCO_3_^−^ that approaches NBCn2 dissociates into CO_3_^=^ and H^+^, with CO_3_^=^ entering the PT cytosol via NBCn2. Virtually all of the newly produced luminal H^+^ titrates the HCO_3_^−^ that NBCn2 moves out of the PT cell. The result is the formation of luminal H_2_O and CO_2_—catalyzed by apical CA IV—and the subsequent influx of H_2_O and CO_2_, again mostly via AQP1, into the cytosol. Once inside the cell, the CO_2_ and H_2_O—catalyzed by CA II—would regenerate HCO_3_^−^ for export into the lumen via NBCn2, and H^+^, which would titrate the incoming CO_3_^=^ to HCO_3_^−^, which would eventually exit the cell via NBCe1-A. Note that, in this scenario, virtually no luminal H^+^ would be left over to titrate B^−^ to HB. In other words, NBCn2 would appear to mediate the pure uptake of NaHCO_3_ without titrating other buffers. AM, apical membrane; BLM, basolateral membrane; CA_Inh_, carbonic anhydrase inhibitor.

The recent discovery that the electroneutral Na/HCO_3_ cotransporter NBCn2 is abundantly present in the apical membrane of the PT [90] is consistent with the idea that this transporter may mediate ~20% of total acid–base traffic across the apical membrane—previously unaccounted for. Although the mechanism of NBCn2 may be the simple cotransport of Na^+^ plus HCO_3_^−^ (Figure 8A), evidence that NBCn2 may in fact be an exchanger [91] is consistent with the idea that NBCn2 exchanges Na^+^ plus CO_3_^=^ for HCO_3_^−^ (Figure 8B). The legend of Figure 8 provides a more detailed summary of these hypothetical processes.

As illustrated in Figure 8A, the role of apical CA IV in the cotransporter model would be to replenish HCO_3_^−^ transported into the cell by NBCn2 and, in the process, generate H^+^ that would, in turn, titrate non-CO_2_/HCO_3_^−^ buffers (i.e., H^+^ + B^−^ → HB) in the tubule lumen. Mathematical modeling predicts that direct HCO_3_^−^ reclamation via NBCn2 would be slightly more efficient energetically than the titration of luminal HCO_3_^−^ by secreted H^+^ [90]. Conversely, mathematical modeling predicts that H^+^ secretion is slightly more efficient energetically than HCO_3_^−^ uptake in titrating luminal non-CO_2_/HCO_3_^−^ buffers like phosphate and NH_3_.

As illustrated in Figure 8B, the role of apical CA IV in the exchanger model would be to dispose of: (a) the H^+^ generated as HCO_3_^−^ dissociates to form CO_3_^=^; and (b) the HCO_3_^−^ transported outward by NBCn2. In this mechanism, virtually none of the H^+^ generated in the lumen would be available to titrate B^−^ to HB. In other words, the exchanger mechanism predicts that NBCn2 would have a single net effect: reclaiming NaHCO_3_.

The important role that CA plays in HCO_3_^−^ reabsorption/H^+^ secretion in renal tubules can be appreciated from experiments with inhibitors. Intraluminal pH measurements have shown that, in the proximal tubule, inhibition of luminal CA causes lumen pH to decrease to a value lower than the equilibrium pH predicted by the Henderson–Hasselbalch equation [92]. Indeed, the model in Figure 7 predicts that inhibition of luminal CA IV would slow the consumption of H^+^ and lead to a fall in luminal pH (pH_Lumen_), as observed. The HCO_3_^−^-uptake model of Figure 8A predicts the opposite. The observation that CA inhibition causes an acid shift in pH_Lumen_ led to two important conclusions: (1) H^+^ secretion is the primary mechanism for acidification of the tubule fluid; and (2) a CA with activity accessible to the tubule fluid is present at the apical membrane [93,94,95,96]. The experiments in proximal tubules perfused in vivo with dextran-bound CA_Inh_—which inhibit only luminal CA—demonstrated that the lumen is in contact with luminal or “membrane-bound” CA and that this CA is necessary for ~80% of HCO_3_^−^ reabsorption [97].

A logical flaw in Conclusion 1 in the previous paragraph is that H^+^ secretion (Figure 7) is not the only mechanism that predicts an acid shift in pH_Lumen_ upon blocking CA IV: The exchanger (i.e., Na^+^ + CO_3_^=^ uptake in exchange for HCO_3_^−^) model in Figure 8B makes the same prediction.

Whereas the pH disequilibrium experiments with CA_Inh_ pointed towards a role of CA IV in HCO_3_^−^ reabsorption, the discovery of the human CA II-deficiency syndrome emphasized the importance of CA II activity in normal renal physiology [23,98]. CA II-deficient patients exhibit renal tubular acidosis, which implicates CA II as an important component of urinary acidification. The development of a CA II-deficient mouse model—which also exhibits a defect in renal acidification—represented another advancement towards understanding the role of renal CA II [99]. Evidence on the involvement of CA II in HCO_3_^−^ reabsorption comes from experiments showing a reduced basolateral HCO_3_^−^ exit [100,101] and a more alkaline intracellular pH [101] in the presence of CA inhibitors.

Another role of renal CA may be facilitation of CO_2_ diffusion across proximal tubule cells [102]. In their elegant study, Schwartz and Burg measured the flux of CO_2_ across isolated perfused rabbit proximal convoluted tubules in the presence of a CO_2_ gradient from bath (i.e., solution facing basolateral membrane) to lumen, or vice versa. To test whether cytosolic and/or membrane-bound CA facilitates the flux of CO_2_ across proximal tubule cells—a mechanism discussed above, in Figure 1, Figure 3, Figure 4—these authors added ACZ to the bath while maintaining a bath-to-lumen CO_2_ gradient. They found that ACZ causes the collected luminal fluid to become more alkaline (because less CO_2_ was arriving in the lumen) and the flux of CO_2_ from bath to lumen to decrease by 55%. These findings (assuming that ACZ can enter PT cells across the basolateral membrane) support the hypothesis that CA facilitates CO_2_ diffusion across proximal-tubule cells. Moreover, following the work of Gutknecht et al. [82] discussed above, Schwarz and Burg observed that the flux of CO_2_ through the UL surrounding the tubule is enhanced when CA is in the bath.

Krahn and Weinstein used mathematical modeling of a rat proximal tubule brush border to investigate the role of CA and of its inhibition on the UL near the brush border [103]. Their modeling approach assumed that the brush border consists of uniform cylinders (representing the villi), equally distributed on a flat surface. By further assuming that the length of the villi is much larger than their width, they ignored the concentration gradients in the radial direction and reduced the problem to one-dimensional in space, with the spatial dimension representing the direction along the long axis of the microvillus. The model includes two compartments: the intra-villous and the intervillous spaces, which are separated by a plasma membrane that contains transporters that move solutes between compartments. The model accounts for the evolution in space (but does not consider time dependence) of concentration profiles of ten solutes (including the components of the CO_2_/HCO_3_^−^ buffer)—both inside and surrounding the microvilli—along the long axis. By reducing the values of the rate constants for the first step of Reaction (2), these authors simulated the inhibition or absence of CA and showed that, in the presence of buffers, CA facilitates the flux of CO_2_ through the UL near the brush border [103].

Finally, we observe that our recent finding of the supra-additive effect of extracellular and intracellular CA on the transmembrane flux of CO_2_ (see Figure 6) is in line with CA-dependent facilitated CO_2_ diffusion across plasma membrane and, more importantly, it may point towards a synergistic role of CA IV and CA II in enhancing the flux of CO_2_ across the apical membrane of the proximal tubule (see Figure 7).

Mathematical modeling has already provided some important insight into some events in Figure 7 and Figure 8A. Distinguishing between the alternate NBCn2 mechanisms—cotransport (Figure 8A) vs. exchange (Figure 8B)—will require more sophisticated modeling approaches, as well understanding the totality of acid–base transport events taking place in the proximal tubule, other nephron segments, and other epithelia engaged in acid–base transport.

### 4.2. The Respiratory System: CO_2_ Removal from the Human Body

**Figure 9:**
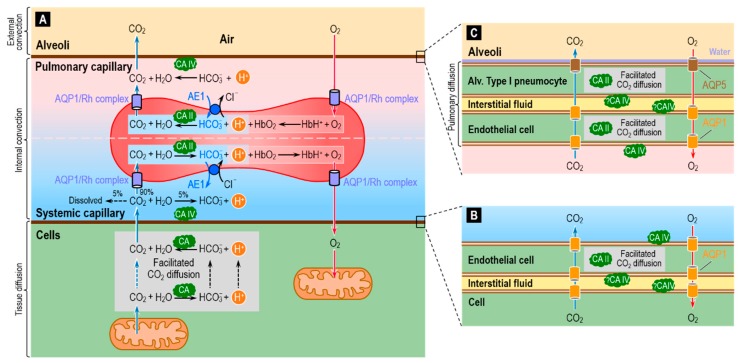
Diagram of the reaction, diffusion and transport processes involved in the removal of the metabolically produced CO_2_ from cells to blood (systemic capillary) and from blood (pulmonary capillary) to the lung (alveoli). (**A**) Of the CO_2_ that diffuses from cells to the systemic capillary, ~10% remains in the blood plasma—as dissolved CO_2_ (~5%), HCO_3_^−^ (~5%) or carbamino compounds (<1%)—whereas ~90% enters the RBCs, almost entirely via the water channel AQP1 [104] or the Rh complex [72]. In the cytosol of the RBCs, most CO_2_ rapidly combines with H_2_O to form HCO_3_^−^ and H^+^, under the catalytic action of CA I (not shown) and CA II. Much of this newly formed HCO_3_^−^ exits via the Cl-HCO_3_ exchanger AE1 [105,106]. A smaller fraction of the incoming CO_2_ covalently reacts with hemoglobin (Hb) to form carbamino-Hb and H^+^, and a tiny fraction remains dissolved in the RBC cytosol. Hb buffers nearly all of the newly formed H^+^ [58,107]. These events all reverse in the pulmonary capillary, where RBCs unload CO_2_ for diffusion into the alveolar space. Another important function of CA in RBCs is augmentation of the association and dissociation of O_2_ with Hb via the Bohr effect. In the systemic capillary, the entry of CO_2_ into RBCs causes their pH to decrease, thereby reducing the Hb-O_2_ binding affinity and favoring O_2_ unloading to the tissue. The formation of carbamino-Hb also favors O_2_ unloading. In the pulmonary capillary, the exit of CO_2_ from the RBCs has the opposite effect, favoring O_2_ loading from the alveoli to the pulmonary capillary. Recent evidence points toward a role of AQP1 and the Rh complex in the diffusion of O_2_ across the RBC membrane [108]. CA IV on the lumen side of the capillary endothelial cells may accelerate the reaction CO_2_ + H_2_O → HCO_3_^−^ + H^+^ in the lumen of systemic capillaries, thereby maximizing flux from tissue to blood, and may accelerate the opposite reaction HCO_3_^−^ + H^+^ → CO_2_ + H_2_O in the lumen of pulmonary capillaries, thereby maximizing CO_2_ flux from blood to alveoli (see Figure 3B). (**B**) Magnification of the different layers of the gas-exchange surface at the systemic capillary. The barrier between the RBC cytosol and capillary lumen actually consists of three plasma membranes—that of the cell that generates the CO_2_, and the two membranes of the endothelial cell—plus the interstitial fluid and cytosol of the endothelial cell. Cytosolic CA would facilitate CO_2_ diffusion through the endothelial cell (see Figure 1) and enhance diffusion across the membranes (see Figure 3B). CA IV may be present on the membranes facing the extremely thin layer of interstitial fluid, where this enzyme could also facilitate CO_2_ diffusion through the interstitial fluid as well as the transmembrane diffusion of CO_2_. Note that AQP1 or other AQPs—depending on the identity of the capillary bed—could provide a low-resistance pathway for the diffusion of CO_2_ and O_2_ across all three of these membranes. (**C**) Magnification of the different layers of the pulmonary capillary-alveolar gas exchange surface. This system is more complex than that in (**B**) because the gases must cross four cells membranes. In addition, AQP5 is present at very high densities in the alveolar side of the alveolar type I pneumocyte [109].

One of the major housekeeping tasks of the human body is the transfer of CO_2_—a waste product of aerobic metabolism—from mitochondria to systemic capillaries, to RBCs that flow to pulmonary capillaries, and then from these RBCs to the alveolar air space, and finally the excretion of this CO_2_ from the body by exhalation. As illustrated in Figure 9A, this process—which along with O_2_ transport is part of external respiration—involves a combination of reactions, diffusion (including facilitated diffusion of CO_2_), transport mediated by membrane proteins, and convection. Diffusion operates over short-distances (i.e., in the neighborhood of the systemic and pulmonary capillaries), and convection dominates over long-distances (i.e., blood flow between systemic and pulmonary capillaries, and air flow between alveoli and environment). The efficient removal of CO_2_ from the body is essential to prevent the build-up of CO_2_ in the intracellular and extracellular compartments, and thereby prevent their acidification.

The widespread presence of CA throughout the respiratory system helps to make the process of CO_2_ removal remarkably efficient. The cytosolic protein CA II is the most abundant form, and RBCs contain high levels of high-activity CA II [37,110]. In addition to CA II, RBCs have even higher levels of the lower-activity CA I. The overall CA activity of RBCs is the highest known of all cell types. It has been estimated that, in RBCs, CAs accelerate the overall reaction CO_2_ + H_2_O ⇌ HCO_3_^−^ + H^+^ by a factor of up to 20,000–25,000 fold [40,72,111,112]. These estimates were recently confirmed by mass spectrometric measurements of the time course of the decay of C^18^O^16^O in human RBCs suspensions—a technique used in combination with a compartmental mathematical model of the reaction and transport processes associated with ^18^O exchange [72,112,113].

Investigators generally agree that blood plasma normally lacks CA activity aside from that attributable to lysed RBCs. However, tissues with high fluxes of CO_2_ into or out of the blood have a component of CA activity that is accessible to the fluid within the capillary lumen. Two cases in point are at least certain systemic capillaries (Figure 9B) and virtually all pulmonary capillaries (Figure 9C). In both cases, the endothelial cells have membrane-bound CA IV at the extracellular luminal surface—particularly well studied in the lung [114,115,116,117,118].

In blood-perfused lungs, RBCs account for ~99% of total CA activity. Of the remaining ~1% (i.e., the CA activity of lung tissue per se), CA II accounts for ~70–90% and CA IV for the rest [110,119,120,121]. Because of the extremely small sizes of the lung cells, it has been challenging to ascertain the cellular and sub-cellular localizations of lung CAs among the various components of the gas-exchange surfaces. Note that the total distance between the pulmonary capillary blood plasma and the alveolar air—the thicknesses of the endothelial cell + the interstitial space + the thin alveolar type I pneumocyte that lines ~90% of the alveolar air sac—is typically ~1 μm [122,123,124]. The consensus already had been that CA II is present in the thicker alveolar type II pneumocytes [110,125], which secrete surfactant, and possibly also in certain endothelial cells [121]. More recently, investigators have detected CA II in alveolar type I pneumocytes [126].

The CA II in systemic capillary endothelial cells (Figure 9B), as well as in alveolar type I pneumocytes and pulmonary capillary endothelial cells (Figure 9C), could in principle facilitate CO_2_ diffusion through the thin layers of cytosol. In addition, aquaporins 1 and 5—known to conduct CO_2_ [71,73,74,75]—could provide a low-resistance pathway for CO_2_ diffusion across the cell membranes.

In additional to its presence on the side of the pulmonary endothelium facing the plasma, CA IV may be accessible to the parenchymal interstitial space [110,114,117], that is, between the pulmonary endothelial cell and the alveolar type I pneumocyte (Figure 9C). Although CA IV may be expressed on the basolateral (i.e., capillary) side of the alveolar epithelium (i.e., the alveolar type I and II pneumocytes), it has not been detected on the apical side (i.e., facing air) [127,128]. One might have thought that CA activity in the alveolar water film would: (a) accelerate CO_2_ efflux from the alveolar type I pneumocyte into the water film and alveolar air; or (b) facilitate CO_2_ diffusion through the water film. Mathematical modeling could provide insight into the requisite lack of evolutionary pressures. Perhaps the presence of AQP5 (CO_2_ as well as H_2_O channels) obviate the need for CA in the water film. Perhaps the water film is so thin that facilitated diffusion is not a substantive issue.

The development of the CA II-deficient mouse model represented an important tool for revealing the location at the cellular level of membrane-associated CA [99,129]. Histochemical examination of lung tissue from the CA II-deficient mouse reveals strong staining that must be due to CA IV in the alveolar epithelium (presumably at the basolateral membrane because CA activity is absent from the apical side [see above]) and possibly some staining in the capillary endothelium (presumably at least at the luminal membrane, where CA activity is detected [see above]). However, because these two epithelia are so thin and so closely opposed, the histochemical analysis could not establish the precise locations of CA IV between the alveolar epithelium and pulmonary capillary endothelium [129].

Understanding the physiological role of CAs in the lungs has been very challenging mainly because of the still-remaining uncertainties on the cellular and subcellular distributions of CA II and CA IV. While it is established that CA II in RBCs is essential for CO_2_ carriage from peripheral tissues to the lungs and for O_2_ carriage via the Bohr effect [42], the roles of CAs in and around the pulmonary capillary endothelium and alveolar type I pneumocytes is unresolved. Mathematical simulations of total or partial CA inhibition in the various components of the gas-exchange surfaces, as well as experiments employing selective inhibitors, have shed light on the function of pulmonary CAs. However, results from in vitro (e.g., using perfused lungs) and in vivo experiments appear to be contradictory. For an in-depth summary of these studies, we refer to Refs. [92,110,121,130]. Here, we briefly review some of the key findings, with special attention to the contribution of mathematical modeling.

As mentioned above, it is well established that membrane-bound CA activity is accessible to the pulmonary capillary lumen. Supporting evidence comes from in vitro and in silico pH disequilibrium studies—in the absence of CA activity accessible to plasma—focused on investigating disturbances of the CO_2_/HCO_3_^−^ equilibrium in blood plasma following addition of CO_2_ to the blood at the systemic capillary beds, or removal of CO_2_ from the blood at the gas-exchange surface. In the 1970s, investigators developed various models to simulate CO_2_ exchange from tissue to blood [131] and from blood to lungs [132,133]. These models treat the blood as a two-compartment system (RBC and plasma), where the relevant chemical reactions (e.g., slow CO_2_ hydration/dehydration) occur. Diffusion within each compartment is instantaneous. The Fick equation [134] describes fluxes of CO_2_ and O_2_ across the RBC membrane and across the endothelium (between blood plasma and either systemic tissue or alveolar air). In Refs. [131,133], the Goldman–Hodgkin–Katz (GHK) equation (i.e., electrodiffusion) [135] describes the movements of HCO_3_^−^ and Cl^−^ (i.e., Cl-HCO_3_ exchange) across the RBC membrane. In Ref. [132], the Fick equation describes the movements of HCO_3_^−^ across the RBC membrane, with the flux of Cl^−^ assumed to be equal but opposite to that of HCO_3_^−^. (Of course, neither the GHK nor the Fick equation can truly mimic the saturation kinetics of the Cl-HCO_3_ exchanger.) These models predicted that, as CO_2_ exchanges with a red cell suspension [131] or with the pulmonary capillary plasma [131,132,133], lack of CA in the extracellular fluid/plasma, causes a pH disequilibrium in that compartment.

In vitro experiments with isolated perfused lungs devoid of blood—a condition important to eliminate the dominant effect of CA in RBCs—and with CA inhibitors of different tissue permeabilities confirmed the above theoretical predictions [136,137,138]. Mathematical simulations predict that one role of the membrane-bound CA at the surface of the pulmonary vascular endothelium is to produce chemical equilibrium within the normal pulmonary-capillary transit time and therefore avoid post-capillary (i.e., in the pulmonary veins and systemic arteries) pH disequilibrium (pH in plasma < pH at equilibrium). In such a disequilibrium state, the continuous slow conversion of HCO_3_^−^ to CO_2_ (see Figure 9A, reaction above the RBC) in the post-capillary plasma leaving the lung would result in a non-physiological situation in which arterial [CO_2_] is higher than alveolar [CO_2_] [110].

Another role of membrane-bound CA IV on the pulmonary capillary endothelium might be to increase the flux of CO_2_ from the blood plasma to the alveolar space [42]. As illustrated by the reaction above the RBC in Figure 9A, CA IV would favor the conversion of plasma HCO_3_^−^ (created in the systemic capillaries from metabolically generated CO_2_) back to CO_2_, thereby maintaining a relatively high plasma [CO_2_] at the gas-exchange interface. Similarly, the CA IV located on the luminal side of the endothelium of systemic capillary (Figure 9A, reactions below the RBC) may facilitate diffusion of CO_2_ from tissue to capillary plasma by keeping plasma [CO_2_] low through rapid conversion of CO_2_ to HCO_3_^−^ [42].

From the above discussion, it should be clear that several authors, as well as others [139,140], believe that pulmonary-capillary CA IV and lung CA in general play a significant role in promoting CO_2_ elimination. However, other authors—citing the very short diffusion distances among the various components of the gas-exchange surfaces between the pulmonary capillary and the alveoli layers—make qualitative arguments against this possibility [121]. One could begin to address this controversy by developing spatially distributed mathematical models that account not only for chemical reactions, but also for diffusion and membrane-protein–mediated transport throughout the system in Figure 9. Future models could also incorporate better kinetic descriptions of the Cl-HCO_3_ exchanger AE1, channels for the conduction of CO_2_ and O_2_, and deformation of the RBCs as they transit along the pulmonary capillaries.

In parallel with our discussion of renal acid–base physiology, mathematical modeling has already provided some important insight into some events in Figure 9A. However, we are still far away from a comprehensive picture of the events taking place inside—and through the membrane of—the RBC, let alone in understanding the events in the microenvironments depicted in Figure 9B,C. Understanding these complex processes will require not only comprehensive wet-lab data, but also sophisticated modeling approaches.

## 5. Conclusions

Since the time of Roughton, we have appreciated that the rapid CA-catalyzed interconversion of CO_2_ and HCO_3_^−^ makes it possible for RBCs to transfer CO_2_ from the systemic tissues to the alveoli (Figure 9). Besides this role in whole-body respiration, CAs are important for a seemingly endless list of processes throughout the body. However, as important as these processes are, merely listing them tends to obfuscate the fundamental principles by which the enzymatic activity of CAs contributes to physiology—contributions that modeling can help to elucidate. Here, we identify three such principles.

First, although we only allude to it in this review, CAs enable the CO_2_/HCO_3_^−^ system to buffer protons on a rapid time scale. For example, a sudden flux of H^+^ into a cell (e.g., H^+^/lactate uptake mediated by the monocarboxylate transporter MCT1) could cause a precipitous—and potentially dangerous—fall in local pH_i_ if buffered only by non-CO_2_/HCO_3_^−^ buffers and the uncatalyzed CO_2_/HCO_3_^−^ reactions. The presence of CA II in the cytosol allows the CO_2_/HCO_3_^−^ system to participate in buffering on a rapid time scale, and thereby minimize pH changes. Related to this issue is the situation depicted in Figure 7, where the challenge of the proximal tubule is to reclaim luminal HCO_3_^−^ even though the PT cell (at least in theory) only has the machinery to: (a) secrete H^+^ into the lumen; and (b) transfer CO_2_ across the apical membrane. Complicating the challenge is that the rapidly flowing luminal fluid (which contains the filtered HCO_3_^−^) is in contact with PT cells for < 1 s. CA IV facilitates HCO_3_^−^ reclamation by rapidly titrating the HCO_3_^−^ to CO_2_—in other words, buffering the secreted H^+.^

Second, CAs can facilitate the transfer of protons between the CO_2_/HCO_3_^−^ buffer system and another buffer system. In the example of Figure 8A, the cell takes up HCO_3_^−^ directly and yet must also titrate B^−^ to HB. Here, the CA IV facilitates the transfer of H^+^ from the CO_2_/HCO_3_^−^ system to another (i.e., B^−^/HB) system.

Third, as outlined in Figure 1, Figure 2, Figure 3, Figure 4 and Figure 5, CAs can maximize gradients—in the bulk solution or across membranes—and thereby facilitate the diffusion of CO_2_, HCO_3_^−^, H^+^, deprotonated buffers (B^−^), and protonated buffers (HB). In such reaction–diffusion systems, the reactions are variations of the interconversions illustrated in Figure 7; Figure 8.

The three fundamental principles just discussed—which underlie the overwhelming majority of CA physiology throughout the different compartments of the body—can be extremely complex, especially when one must consider reactions involving not only HCO_3_^−^ but CO_3_^=^, or when the system contains multiple non-CO_2_/HCO_3_^−^ buffers. In such complex systems, mathematical modeling can be invaluable for assessing the relative importance of different components or pathways (e.g., H^+^ secretion vs. HCO_3_^−^/CO_3_^=^ uptake in Figure 7 and Figure 8). The models also help investigators formulate hypotheses, and make predictions that the investigators can test in wet-lab experiments. For example, what would be the predicted effect (based on mathematical simulations) of blocking or knocking out a particular CA or acid–base transporter?

A variation on the complexity issue in the previous paragraph is another important problem in biology: dealing with missing data. In the model of Figure 9, we are still uncertain about whether particular CAs are present and, if so, where? Again, simulations based on specific hypotheses can make predictions that are testable in the wet lab (e.g., based on inhibitors, knockouts). For example, one could use modeling to explore how, in principle, different spatial expression patterns for CAs and AQPs could optimize gas exchange (e.g., enhancing CO_2_ removal) in health, or compromise it in disease.

Another variation on the missing-data theme is to use simulations to infer data that one cannot easily measure. Consider, for example, an investigator who monitors pH_i_ while exposing a cell to CO_2_/HCO_3_^−^. Even a relatively simple compartmental model could provide valuable insight into how [CO_2_] and [HCO_3_^−^]—two parameters not directly measured—change with time in the cytosol [141]. With sophisticated spatially distributed mathematical models, one could get insight into how pH, [CO_2_], and [HCO_3_^−^] change with time at different depths beneath the cell membrane, and how the spatiotemporal profile may change with the introduction of CA IV at the outer surface of the membrane and/or CA II in the bulk intracellular fluid. Even more sophisticated mathematical models—together with appropriate computational methods—could begin to account for more complex, but still not entirely realistic, geometries like those in Figure 9.

In summary, mathematical models involving CA and CO_2_/HCO_3_^−^ can be an invaluable complement to wet-lab experiments by providing unprecedented insight into physiological mechanisms as well as guidance in both data interpretation and the design of future experiments.
